# Visible‐Light‐Induced Hydrogen Generation from Mixtures of Hydrogen Boride Nanosheets and Phenanthroline Molecules

**DOI:** 10.1002/advs.202405981

**Published:** 2024-09-13

**Authors:** Jumpei Takeshita, Hayato Tsurugi, Andi Mauliana, Akira Yamaguchi, Takahiro Kondo, Masahiro Miyauchi

**Affiliations:** ^1^ Department of Materials Science and Engineering School of Materials and Chemical Technology Tokyo Institute of Technology Meguro‐ku Tokyo 152–8552 Japan; ^2^ Department of Applied Chemistry Graduate School of Engineering Osaka University Suita Osaka 565–0871 Japan; ^3^ Innovative Catalysis Science Division Institute for Open and Transdisciplinary Research Initiatives (ICS‐OTRI) Osaka University Suita Osaka 565–0871 Japan; ^4^ Department of Materials Science Institute of Pure and Applied Sciences University of Tsukuba Tsukuba 305–8573 Japan; ^5^ The Advanced Institute for Materials Research Tohoku University Sendai Miyagi 980–8577 Japan; ^6^ Tsukuba Research Center for Energy Materials Science Institute of Pure and Applied Sciences and R&D Center for Zero CO_2_ Emission Functional Materials University of Tsukuba Tsukuba 305–8573 Japan

**Keywords:** hydrogen boride, hydrogen generation, nanosheet, phenanthroline, visible light

## Abstract

Hydrogen boride (HB) nanosheets are recognized as a safe and lightweight hydrogen carrier, yet their hydrogen (H_2_) generation technique has been limited. In the present study, nitrogen‐containing organic heterocycles are mixed with HB nanosheets in acetonitrile solution for visible‐light‐driven H_2_ generation. After exploring various nitrogen‐containing heterocycles, the mixture of 1,10‐phenanthroline molecules (Phens) and HB nanosheets exhibited significant H_2_ generation even under visible light irradiation. The quantum efficiency for H_2_ generation of the mixture of HB nanosheets and Phens is 0.6%. Based on spectroscopic and electrochemical analyses and density functional theory (DFT) calculations, it is determined that radical species generated from Phens with electrons and protons donated by HB nanosheets are responsive to visible light for H_2_ generation. The HB nanosheets/Phens mixture presented in this study can generate H_2_ using renewable energy sources such as sunlight without the need for complex electrochemical systems or heating mechanisms and is expected to serve as a lightweight hydrogen storage/release system.

## Graphical abstract

The mixture of 1,10‐phenanthroline molecules (Phens) and HB nanosheets in acetonitrile solution generates gaseous hydrogen under visible light irradiation. Phens with electrons and protons donated by HB nanosheets are responsive to visible light for hydrogen generation.

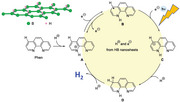

## 1 Introduction

Two dimentional (2D) nanomaterials, such as graphene, have garnered significant attention in research and development for various functional applications.^[^ ^1^ ^]^ While many researchers have tried to explore new classes of 2D nanomaterials beyond graphene, theoretical predictions suggest that boron‐based 2D materials may exhibit unique mechanical and electronic properties.^[^ ^2^ ^]^ Recently, transition metal boride‐based 2D materials have been reported.^[^ ^3^ ^]^ In addition to metal borides, single‐layered boron nanosheets (borophenes) without metal components were successfully grown on a silver substrate through a dry process.^[^ ^4^ ^]^ Based on these experimental studies, other boron‐based 2D materials, such as hydrogenated borophenes (borophanes), have been reported to form by exposing a borophene film to an atomic hydrogen source.^[^ ^5^ ^]^ Borophanes, also called hydrogen boride (HB) nanosheets, have been the subject of theoretical studies indicating their distinct mechanical and electronic properties.^[^ ^6^ ^]^ Recently, Nishino et al. successfully synthesized free‐standing HB nanosheets using a facile wet chemical method.^[^ ^7^ ^]^ This pioneering synthetic work unveiled intriguing properties of HB nanosheets, including solid acidity,^[^ ^8^ ^]^ catalytic carbon dioxide conversion,^[^ ^9^ ^]^ and their reducibility.^[^ ^10^ ^]^ HB nanosheets are also used as a hydrogen storage material^[^ ^11^ ^]^ due to their high gravimetric hydrogen capacity of 8.5 wt%, which surpasses that of many other hydrogen carriers such as metal hydrides or organic hydrides.^[^ ^12^ ^]^ However, for practical application as a hydrogen storage material, the hydrogen generation technique from HB nanosheets by an external stimulus is crucial. Previous studies have reported that gaseous hydrogen molecules (H_2_) can be generated from HB nanosheets by heat,^[^ ^7^ ^]^ electrochemical bias,^[^ ^13^ ^]^ and ultraviolet light irradiation.^[^ ^14^ ^]^ Unfortunately, these methods for H_2_ generation require significant energy input. Therefore, the utilization of a renewable energy source like sunlight is desired for H_2_ generation from HB nanosheets. However, HB nanosheets have not shown sensitivity to visible light for H_2_ generation by themselves.^[^ ^14^ ^]^

In this study, we modified HB nanosheets by blending them with organic molecules for visible‐light‐induced H_2_ generation. Among various organic molecules, we have focused on nitrogen‐containing heterocycles like 1,10‐phenanthroline (Phen). These nitrogen‐containing heterocycles are optically transparent in the visible light region, yet some of their radical cations exhibit visible light absorption.^[^ ^15^ ^]^ It is noted that the HB nanosheets possess reducibility^[^ ^10a^ ^]^ and proton donation properties.^[^ ^16^ ^]^ Therefore, it is expected that nitrogen‐containing heterocycles will exhibit coloration upon mixing with HB nanosheets. In this study, HB nanosheets were mixed with nitrogen‐containing heterocycles in an acetonitrile solution, and the hydrogen generation properties of these dispersed mixtures were evaluated under visible light irradiation. Through screening various heterocycles to identify the optimum molecule for H_2_ generation, it was shown that the mixture of 1,10‐phenanthroline molecules (Phens) and HB nanosheets significantly exhibited visible‐light‐induced H_2_ generation. In this study, we discuss the mechanism of H_2_ generation under visible light through density functional theory (DFT) calculations, spectroscopic analyses, and electrochemical studies.

## 2 Results and Discussion

**Figure 1:**
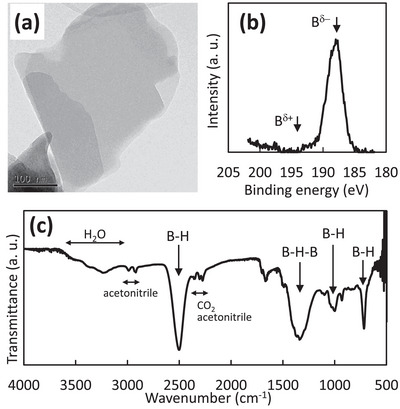
a) TEM image, b) XPS result, and c) FT‐IR spectrum of the synthesized HB nanosheets.

HB nanosheets were synthesized via a wet‐chemical exfoliation process using a magnesium diboride (MgB_2_) precursor, in combination with a proton‐exchange resin in an acetonitrile solution.^[^ ^7^ ^]^ A detailed experimental method is provided in the Experimental Section. **Figure** **1**a shows the transmission electron microscopy (TEM) image of HB nanosheets, revealing thin sheet‐like structures. We also conducted the atomic force microscope (AFM) analysis on HB nanosheets coated on an atomically flat mica substrate (Figure S3 in Supporting Information). The thickness of HB nanosheets was a few nanometers, suggesting their multi‐layered structures. In Figure 1b, the X‐ray photoelectron spectroscopy (XPS) of a boron 1s orbital exhibits a strong peak around 187 eV, indicative of negatively charged boron species (B^δ−^), according to the previous reports.^[^ ^7^, ^17^ ^]^ Additionally, our Supporting Information includes the XPS spectrum of magnesium 2p orbital for HB nanosheets (Figure S4, Supporting Information), where no obvious peak of magnesium is observed. We also conducted the inductively coupled plasma (ICP) analysis of HB nanosheets with two different batches to check if magnesium ions exist as an impurity. However, the content of Mg was less than 0.033 wt% versus B (0.015 mol% versus B), indicating successful ion exchange from magnesium ions to protons within the 2D hexagonal network of boron. Figure 1c shows the Fourier transform infrared spectroscopy (FT‐IR) results of HB nanosheets. Although signals from the acetonitrile solvent and gaseous carbon dioxide are present, peaks corresponding to B–H stretching (2500 cm^−1^), B–H bending (715 and 1000 cm^−1^), and B–H–B bonding (1360 cm^−1^) are observed in the HB nanosheets which is consistent with previous reports.^[^ ^7^, ^18^ ^]^ Our previous thermal desorption spectroscopy (TDS) analysis determined the elemental ratio of the HB nanosheets as H:B = 1:1.02 ± 0.18. These results strongly suggest the following ion exchange process,^[^ ^7^ ^]^

(1) $$\mathrm{Mg}B_{2}+2H^{+}\to Mg^{2+}+2\mathrm{HB}$$

**Figure 2:**
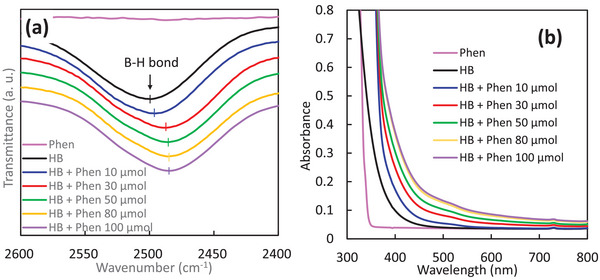
a) FT‐IR spectra and b) UV–vis absorption spectra in 5 mL acetonitrile solution. The amount of HB nanosheets used was 237 µmol for both FT‐IR and UV–vis experiments.

In our investigation to determine the optimal organic molecule for visible‐light‐induced H_2_ generation from the mixture of HB nanosheets with various nitrogen‐containing heterocycles, we explored 11 samples, as listed in Note S1 of our Supporting Information. We hypothesized that nitrogen‐containing heterocycles would be suitable candidates for modification with HB nanosheets due to the visible light absorption by their radical cations.^[^ ^15^ ^]^ Here, we particularly discuss the characterization results of the optimized mixture for H_2_ generation, namely Phens and HB nanosheets. **Figure** **2**a shows the FT‐IR spectra of the B–H bond for Phen‐modified HB nanosheets with different concentrations of Phens. While no peak corresponding to Phens is observed in this region (pink line in Figure 2a), the B–H bond signal is evident in samples containing HB nanosheets. It is noted that the peak position of the B–H bond shifts towards a smaller wavenumber upon modification with Phens. These results indicate hydrogen‐bonding interactions between HB nanosheets and Phens, wherein nitrogen atoms of Phens are probably acting as Brønsted basic sites toward the acidic protons of HB nanosheets, forming N–H bonds. The peak shift becomes more pronounced with a higher amount of Phens, saturating at an additional 80 µmol of Phens.

Figure 2b shows the UV–vis absorption spectra of the mixtures of HB nanosheets and Phens dispersed in an acetonitrile solution. Phens without HB nanosheets only absorbed UV light below 350 nm, while HB nanosheets without Phens absorbed photons shorter than 450 nm. In contrast to both pristine Phens and pristine HB nanosheets, mixtures of HB nanosheets and Phens exhibited two steps of visible light absorption: one starting from 700 nm and another from 550 nm. The intensity of absorbance depended on the amount of Phens, and absorption in the visible light region saturated around 80 µmol of Phens added, similar to the trend observed in the peak shift of the B–H bond in FT‐IR spectra. These results indicate that the visible light absorption of mixtures originated from the interaction between HB nanosheets and Phens.

**Figure 3:**
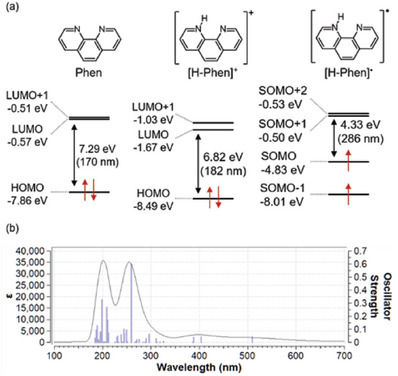
Ground state electronic structures of Phen, protonated Phen ([H‐Phen]^+^) and singly‐reduced protonated Phen ([H‐Phen]•) a). Panel b) shows the calculated absorption spectrum of singly‐reduced protonated Phen. All calculations were carried out using Gaussian 16 program version C.01 with CAM‐B3LYP functional/GD3‐BJ at the 6–311+G(d,p) basis set in SMD (CH_3_CN). The detailed calculation method is described in our Note S3, Supporting Information.

Here, we delve into the origin of the visible light absorption observed in the mixture of HB nanosheets and Phens using DFT calculations. Previous reports indicated that Phens were optically transparent, but their radical forms exhibited coloration.^[^ ^15a^ ^]^ We calculated the electronic structures of Phen and its radical species, namely Phen with a proton and Phen with a proton and an electron, as depicted in **Figure** **3**. After the mono‐protonation of Phen, its highest occupied molecular orbital (HOMO) and lowest unoccupied molecular orbital (LUMO) energy levels shifted towards negativity, facilitating electron acceptance (panel a). In the case of Phen with a proton and an electron, the HOMO‐LUMO gap narrowed compared to pristine Phen. Figure 3b shows the calculated optical absorption spectrum of Phen with a proton and an electron. Similarly to the experimental optical absorption spectra of the HB nanosheets and Phens mixtures shown in Figure 2b, two steps of visible light absorption are evident in the calculated spectrum. These DFT calculations strongly suggest that the visible light absorption originates from the radical species of Phens, which was in situ formed by the protons and electrons donated by HB nanosheets.

**Figure 4:**
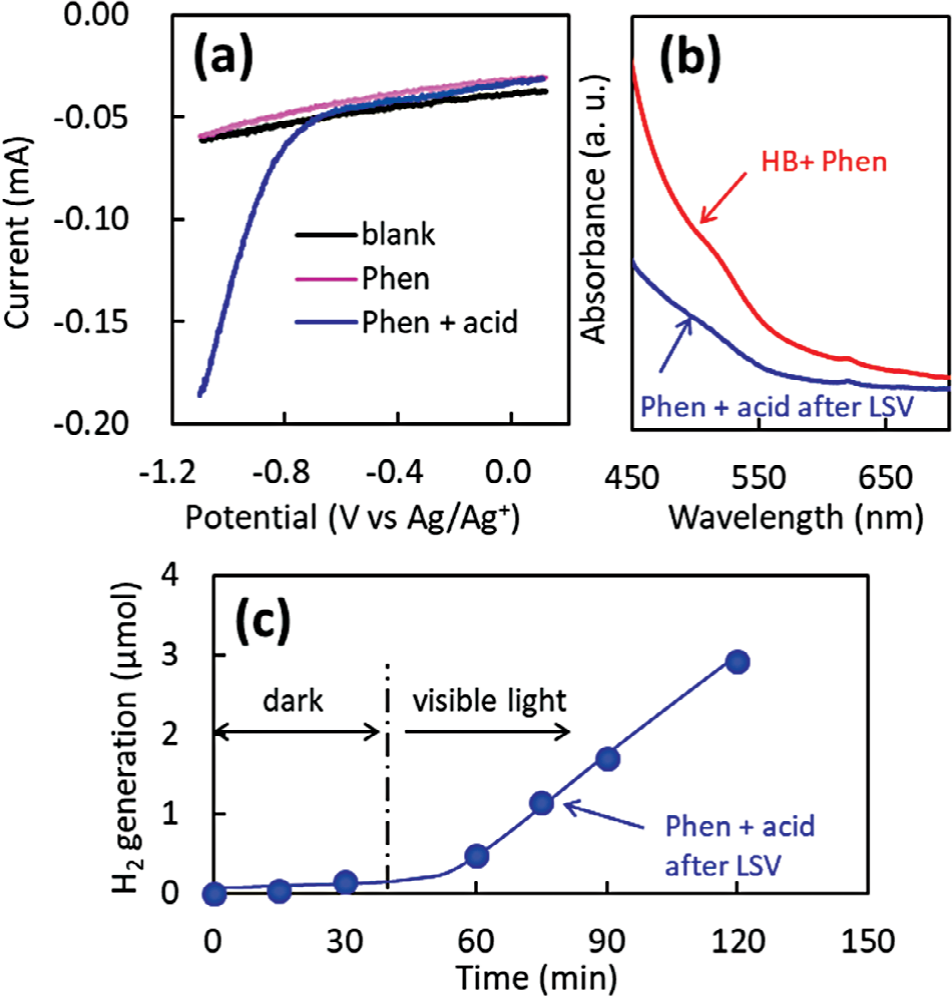
Linear sweep voltammetry (LSV) of Phen under acidic condition a), where 1 mmol of Phens and 0.1 mmol of hydrochloric acid were added into 10 mL of acetonitrile solution with 1 mmol of tetrabutylammonium hexafluorophosphate (TBAPF_6_) as the supporting electrolyte. Platinum plates served as working and counter electrodes, while an Ag/Ag^+^ electrode was used as the reference electrode. Panel b) shows the UV–vis absorption spectra for Phens after the electrochemical reduction (blue line) and the simple mixture of HB nanosheets and Phens (red line). Panel c) shows the H_2_ generation property under visible light (>470 nm) irradiation from Phens after electrochemical reduction in acidic conditions.

To confirm that the visible light absorption originates from Phens with protons and electrons, we tried to produce these radicals by electrochemical reduction of Phens under acidic conditions without the addition of HB nanosheets. Phens were dissolved in an acetonitrile solution with electrolytes, and linear sweep voltammetry (LSV) was performed, as shown in **Figure** **4**a. In the case of Phens with acid, a strong reduction current was observed under the cathodic bias application. After the reduction reaction, the UV–vis spectrum of the solution was recorded, shown in Figure 4b. Phens reduced under acidic conditions exhibited visible light absorption, similar to the case observed in the mixture of Phens and HB nanosheets. These results strongly imply that the visible light absorption in the mixture of HB nanosheets and Phens originates from the radical species of Phens with protons and electrons. Furthermore, Figure 4c shows the H_2_ generation property under visible light irradiation for the Phens after electrochemical reduction in acidic conditions. It is noted that gaseous H_2_ molecules were generated from the radical species of Phens under visible light irradiation. These results indicate that the electron transition from singly occupied molecular orbital (SOMO) to SOMO+1 and SOMO+2 by the visible light excitation induces H_2_ generation from Phens radicals. Additionally, these results imply that H_2_ molecules can be generated from the mixture of HB nanosheets and Phens under visible light irradiation.

While we particularly demonstrated the characterization results of the mixture of HB nanosheets and Phens, we investigated the H_2_ generation properties of other nitrogen‐containing heterocycles, as shown in Figure S5, Supporting Information. Among various nitrogen‐containing heterocycles screened, Phens exhibited the highest H_2_ generation rate among various molecules listed in Figure S5, Supporting Information. The electronic structures of these heterocycles were analyzed using DFT calculations, as illustrated in Figure S6 in our Supporting Information. Phens with electron‐donating substituents such as methyl, methoxy, and amino groups were good organic bases for accepting protons. Conversely, electron transfer to the protonated Phens was rather difficult compared with Phen due to the higher HOMO energy levels; electron‐deficient Phens with chloro, carbonyl, and extended π‐motif were weak organic bases for protonation by HB nanosheets, thus resulting in decreasing the concentration of the protonated form. Based on the different proton and electron affinities, Phens (1,10‐phenanthroline) exhibited the highest H_2_ release property among the various nitrogen‐containing heterocycles in the present study.

**Figure 5:**
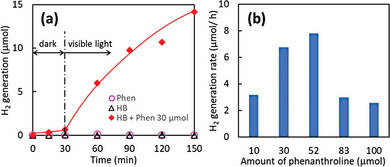
H_2_ generation properties under visible light (>470 nm) irradiation. a), and dependence of H_2_ generation rates on Phens amount for 2 h b).

Here, we demonstrate detailed H_2_ production properties from the mixture of HB nanosheets and Phens under visible light irradiation. **Figure** **5**a shows the time course of H_2_ generation under visible light irradiation. H_2_ molecules were generated under visible light irradiation from the mixture of HB nanosheets and Phens, while negligible H_2_ generation was observed from pristine HB nanosheets and pristine Phens. These results indicate that the radical species of Phens are excited under visible light, leading to the generation of H_2_ molecules, similar to the case of the electrochemically reduced Phens as shown in Figure 4c. A small amount of H_2_ was generated even under dark conditions from HB + Phen and Phen + acid after LSV, presumably owing to the mechanical stimulation by stirring. Figure 5b shows the effect of Phens amount on the H_2_ generation properties under visible light. The optimal amount of Phens for H_2_ generation ranged between 30–60 µmol. As shown in Figure 2, the shift of FT‐IR signals and visible light absorption saturated over 80 µmol. These results indicate that the overloading of Phens suppresses H_2_ generation, probably due to the capture of acidic protons on HB nanosheets by excessive amounts of Phen.

**Figure 6:**
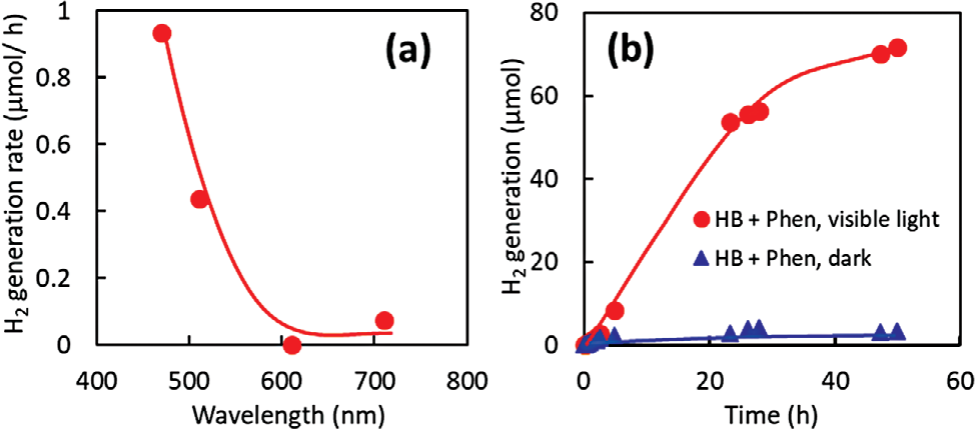
a) Action spectrum of the HB nanosheets and Phens mixture for H_2_ generation using optical short‐wavelength cutoff (long wavelength pass) filters, and b) long‐term H_2_ generation properties of the mixture of HB nanosheets (237 µmol) and Phens (10 µmol) under visible light (>470 nm) irradiation (red circles) and dark conditions (blue triangles).

The action spectrum of H_2_ generation from the mixture of HB nanosheets and Phens was evaluated using optical cutoff filters (**Figure** **6**a). Gaseous H_2_ molecules were generated under light irradiation with wavelengths shorter than 600 nm. The trend of the action spectrum aligns with the absorption spectrum shown in Figures 2b and 4b. These results further indicate that the visible light absorption of the radical species of Phens is the primary driver of H_2_ generation.

Figure 6b shows the long‐term H_2_ generation properties under visible light. The amount of generated H_2_ after 50 h of visible light irradiation was 72 µmol. The amount of Phens used in this experiment was 10 µmol. If the generated H_2_ molecules were to originate from the hydrogen atoms of Phens themselves, the expected H_2_ generation would be 40 µmol, which is significantly smaller than the actual H_2_ generation from the mixture of HB nanosheets and Phens. These results indicate that the protons donated from HB nanosheets contribute to the catalytic H_2_ production from Phens. The quantum efficiency for H_2_ generation per absorbed photon number was 0.6%. The calculation method of the quantum efficiency is described in Note S2, Supporting Information. HB nanosheets have terminal B─H bonds and bridging B─H─B bridging bonds,^[^ ^18a^ ^]^ and both or one of them contribute to H_2_ generation from the mixture of HB nanosheets and Phens. We conducted the TEM observation of the mixture of HB nanosheets and Phens after visible light irradiation (Figure S7 in Supporting Information), and the 2D sheet‐like structure was maintained even after light irradiation.

**Figure 7:**
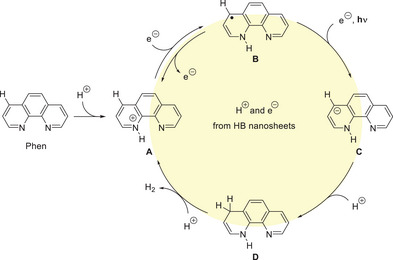
Speculated mechanism of H_2_ generation from the mixture of HB nanosheets and Phens under visible light irradiation.

The role of HB nanosheets is the proton and electron source to Phens. Visible light absorption originates in Phens with protons and electrons. In this manner, the mixture of HB nanosheets and Phens is similar to the organic molecule‐based photocatalysts such as metal complexes rather than solid‐state semiconductor‐based photocatalysts. **Figure** **7** shows the proposed mechanism of H_2_ generation from the mixture of HB nanosheets and Phens under visible light irradiation. HB nanosheets donate a proton and an electron sequentially into Phen, leading to the formation of the protonated form **A** ([H‐Phen]^+^) and its radical **B** ([H‐Phen]•), which absorbs visible light. Under the visible light irradiation, **B** undergoes an additional one‐electron reduction to form carbanion **C,** followed by protonation by HB nanosheets, resulting in the formation of dihydroPhen **D** which serves as an organic hydride source for generating H_2_ upon reaction with acidic protons of HB nanosheets. During the process, HB nanosheets are oxidized, and H_2_ generation is terminated when protons and/or electrons in HB nanosheets are depleted. The addition of protons and electrons to HB nanosheets is crucial for sustainable H_2_ generation. It is intriguing that Phens radicals can be easily produced by a simple mixture of HB nanosheets and Phens, in contrast to the electrochemical method shown in Figure 4, which requires a complex electrode system. This study is valuable for the development of an energy‐efficient H_2_ generation system on a simple mixture of HB nanosheets and nitrogen‐containing organic molecules.

## 3 Conclusion

Gaseous H_2_ molecules were generated from a mixture of HB nanosheets and Phens dispersed in acetonitrile solution under visible light irradiation. HB nanosheets provided protons and electrons to Phens, forming radical species of Phens. The electrochemical analysis and DFT calculations confirmed that the radical species of Phens absorbed visible light and further generated H_2_ molecules under visible light irradiation. Among various nitrogen‐containing heterocycles screened for efficient H_2_ generation, Phens emerged as the most effective molecule for modification with HB nanosheets, with a quantum efficiency for H_2_ generation of 0.6%. The simple mixture of HB nanosheets and Phens facilitates the development of a visible‐light‐sensitive H_2_ generation system without the need for a complicated electrochemical device, making it useful for hydrogen storage/generation using renewable energy such as sunlight.

## 4 Experimental Section

### Synthesis of HB Nanosheets

Similar to the previous report,^[^ ^7^ ^]^ MgB_2_ powder (500 mg) was added to 150 mL of acetonitrile containing an ion exchange resin and stirred at room temperature under a nitrogen atmosphere for three days. The resulting dispersion was filtered to remove unreacted MgB₂ and the ion exchange resin and then dried under vacuum to obtain the final powder product of the HB nanosheets. All preparation and characterization procedures were performed inside a glove box under a nitrogen atmosphere to prevent oxidation of the sample.

### Preparation of Mixtures of HB Nanosheets and Nitrogen‐containing Heterocycles

HB nanosheets (237 µmol, 2.8 mg) and 10 µmol of nitrogen‐containing heterocycle molecules (4.2 mol% with respect to HB nanosheets) were added to 5 mL of acetonitrile solution. 11 nitrogen‐containing heterocycles were used in the present study as shown in the Note S1, Supporting Information.

### Characterization

Transmission electron microscopy (TEM) was performed using a JEM‐2100F TEM/STEM instrument (JEOL Ltd., Japan) operated at an electron beam acceleration voltage of 200 kV. X‐ray photoelectron spectroscopy (XPS) measurements were performed using a JPS 9010 TR (JEOL, Ltd., Japan) instrument with an Al Kα X‐ray source (1486.6 eV). The samples for the XPS analyses were fixed on carbon tape. The absolute binding energy was calibrated based on the carbon (C‐1s) orbital peaks. Fourier‐transformed infrared (FT‐IR) spectra were recorded using an FT/IR‐6100 (JASCO, Co., Ltd., Japan) with an attenuated total reflection (ATR) unit. UV–visible (UV–vis) absorption spectra were recorded using a spectrophotometer (V‐670, JASCO, Co., Ltd., Japan). For an atomic force microscope (AFM) measurement, HB nanosheets were coated on an atomically flat mica substrate by spin coating using the solution of HB nanosheets dispersed in acetonitrile. The spin coating was conducted at 2000 rpm for 20 s. The AFM image was obtained in the dynamic mode using SPM‐9700 (Shimadzu Corp., Kyoto, Japan) with a silicon cantilever. Inductively coupled plasma (ICP) optical emission spectroscopy was performed using a 5100 VDV ICP‐OES instrument (Agilent Technologies). For ICP analysis, HB nanosheets were dissolved in nitric acid solution at 200 °C for 12 h.

### Electrochemical Production of Phens with Electrons and Protons

The electrochemical measurements were performed using a single‐compartment closed cell. Platinum plates were used as working and counter electrodes, while an Ag/Ag^+^ electrode was used as a reference electrode. Phens (1 mmol) were dissolved in 20 mL of acetonitrile with 10 mmol of TBAPF_6_ (97.0%, FUJIFILM Wako Pure Chemical Industries Ltd., Osaka, Japan) and 0.1 mmol of hydrochloric acid. The electrolyte with Phens was prepared in a glove box to prevent oxygen and/or water contamination. Electrochemical cathodic bias was applied using a potentiostat (HZ‐7000; HOKUTO DENKO, Co., Ltd., Japan). UV–visible (UV–vis) absorption spectra after cathodic bias application were recorded using a spectrophotometer (V‐670, JASCO, Co., Ltd., Japan). H_2_ concentration in the headspace of a reactor was detected using a gas chromatograph equipped with a thermal conductivity detector (GC‐2014AT; Shimadzu, Co., Ltd., Japan).

### H_2_ Generation Property

The experimental setup for light‐induced H_2_ release is shown in Figure S1. The dispersed HB nanosheets and heterocycles mixture was introduced into a cell and irradiated with visible light using a xenon lamp through an optical long‐wavelength pass filter (short cutoff) at 470 nm. The spectrum of a xenon lamp is shown in the Figure S2, Supporting Information. Its integrated light intensity was 33.7 mW cm^−2^. Argon (Ar) gas was introduced into the headspace of the reactor, and the H_2_ concentration in the headspace was measured using a gas chromatograph equipped with a thermal conductivity detector (TCD). The spectrum of the light source (incident photon) was recorded by a spectroradiometer (USR‐45, Ushio Ltd.). The quantum efficiency (*QE*) was calculated by considering the two electrons process for H_2_ generation (Detailed calculation method was shown in the Note S2, Supporting Information.

## Conflict of Interest

The authors declare no conflict of interest.

## Supporting information



Supporting Information

## Data Availability

The data that support the findings of this study are available from the corresponding author upon reasonable request.
