# Supplementary material for: Visible‐Light‐Induced Hydrogen Generation from Mixtures of Hydrogen Boride Nanosheets and Phenanthroline Molecules
Source: Adv Sci (Weinh). 2024 Sep 13;11(42):2405981. doi: 10.1002/advs.202405981 (PMC11558089; doi:10.1002/advs.202405981)
Supplement: Supplementary file 1 — Supporting Information [file ADVS-11-2405981-s001.pdf]

ADVANCED SCIENCE
Open Access

Supporting Information

for *Adv. Sci.*, DOI 10.1002/advs.202405981

Visible-Light-Induced Hydrogen Generation from Mixtures of Hydrogen Boride Nanosheets and Phenanthroline Molecules

Jumpei Takeshita, Hayato Tsurugi, Andi Mauliana, Akira Yamaguchi, Takahiro Kondo* and Masahiro Miyauchi**

Supporting Information

Visible-Light-Induced Hydrogen Generation from Mixtures of Hydrogen Boride Nanosheets and Phenanthroline Molecules

Jumpei Takeshita,[a] Hayato Tsurugi,*[b,c] Andi Mauliana,[a] Akira Yamaguchi,[a]

Takahiro Kondo, *[d,e,f] Masahiro Miyauchi *[a]

[a] Department of Materials Science and Engineering, School of Materials and Chemical Technology, Tokyo Institute of Technology, Meguro-ku, Tokyo 152-8552, Japan.

[b] Department of Applied Chemistry, Graduate School of Engineering, Osaka University, Suita, Osaka 565-0871, Japan.

[c] Innovative Catalysis Science Division, Institute for Open and Transdisciplinary Research Initiatives (ICS-OTRI), Osaka University, Suita, Osaka 565-0871, Japan

[d] Department of Materials Science, Institute of Pure and Applied Sciences, University of Tsukuba, Tsukuba 305-8573, Japan.

[e] The Advanced Institute for Materials Research, Tohoku University, Sendai, Miyagi 980-8577, Japan.

[f] Tsukuba Research Center for Energy Materials Science, Institute of Pure and Applied Sciences and R&D Center for Zero CO_2 Emission Functional Materials, University of Tsukuba, Tsukuba 305-8573, Japan.

Corresponding Authors,
H. Tsurugi, tsurugi@chem.eng.osaka-u.ac.jp
T. Kondo, takahiro@ims.tsukuba.ac.jp
M. Miyauchi, mmiyauchi@ceram.titech.ac.jp

Content

Note 1. Nitrogen heterocycles used in this study.

Note 2. Quantum efficiency for H_2 generation.

Figure S1. Experimental setup for the evaluation of H_2 generation under visible light irradiation.

Figure S2. Spectrum of the visible light source (500 W Xe lamp with an optical cutoff filter).

Figure S3. AFM image of HB nanosheets coated on an atomically flat mica substrate.

Figure S4. XPS spectrum of the magnesium 2p orbital for HB nanosheets.

Figure S5. H_2 generation properties of various mixtures under visible light irradiation and molecular structures of nitrogen containing heterocycles

Figure S6. Electronic structure of various heterocycles with a proton and an electron by DFT calculations.

Figure S7. TEM images of HB+Phen before (a) and after visible light irradiation (b).

Note 3. DFT calculations method.

Note 1. Nitrogen heterocycles used in this study

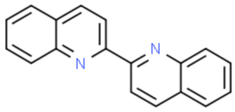

① 2,2'-biquionoline

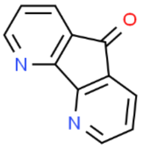

② diazafluorene

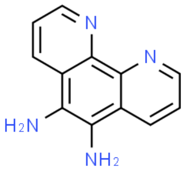

③ 5,6-diamino-1,10-phenanthroline

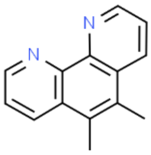

④ 5,6-dimethyl-1,10-phenanthroline

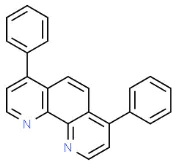

⑤ bathophenanthroline

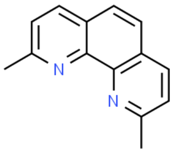

⑥ neocuproine

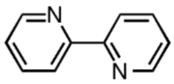

⑦ 2,2'-bipyridyl

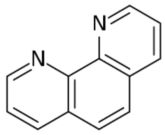

⑧ 1,10-phenanthroline

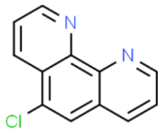

⑨ 5-chrolo-1,10-phenanthroline

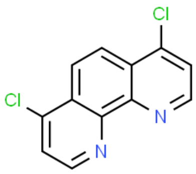

⑩ 4,7-dichloro-1,10-phenanthroline

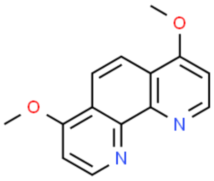

⑪ 4,7-dimethoxy-1,10-phenanthroline

Note 2. Quantum efficiency for H_2 generation

The quantum efficiency (*QE*) was calculated using the following equation by considering the two electrons process for H_2 generation.

$$QE = \frac{Rate\ of\ used\ electron\ number\ [s^{-1}]}{Absorbed\ photon\ number\ [s^{-1}]} = \frac{H2\ generation\ [mol\ s^{-1}] \times 2 \times N_A[mol^{-1}]}{\sum_{\lambda=200}^{\lambda=800}\{Absorption \times Incident\ Photon\}}$$

$$= \frac{H2\ generation\ [mol\ s^{-1}] \times 2 \times N_A[mol^{-1}]}{\sum_{\lambda=200\ nm}^{\lambda=800\ nm}\left\{(Absorption) \times \frac{(E[\mu W \cdot cm^{-2}] \times 10^{-6})}{h[j \cdot s] \times c[m \cdot s^{-1}]/(\lambda[nm] \times 10^{-9})}\right\} \times A[cm^2]}$$

$Absorption\ was\ recorded\ by\ an\ UV - Vis\ spectroscopy$

$N_A[mol^{-1}]$: Avogadro number (6.02×10^{23})

$E[\mu W \cdot cm^{-2}]$: light energy when the wavelength is λ nm, recorded by a spectroradiometer

$h[j \cdot s]$: Planck constant($= 6.62 \times 10^{-34}$)

$c[m \cdot s^{-1}]$: Speed of light ($= 3.0 \times 10^{8}$)

$\lambda[nm]$: Wavelength

$A[cm^2]$: Light irradiated area

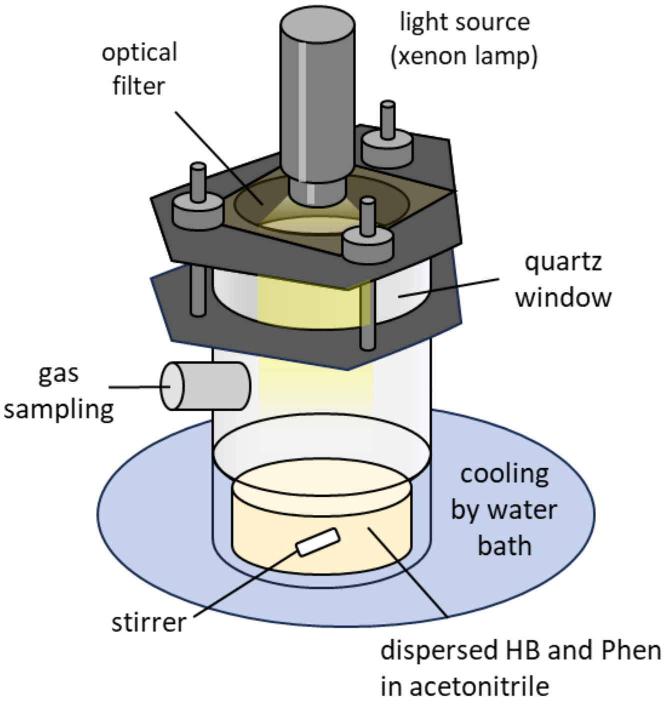

Figure S1. Experimental setup for the evaluation of H_2 generation under visible light irradiation.

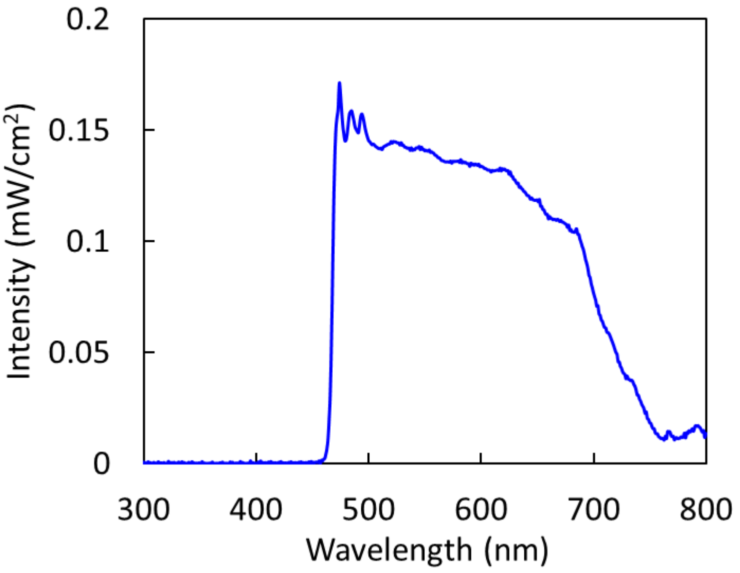

Figure S2. Spectrum of the visible light source (500 W Xe lamp with optical cutoff filter shorter than 470 nm). The integrated light intensity was 33.7 mW /cm^2.

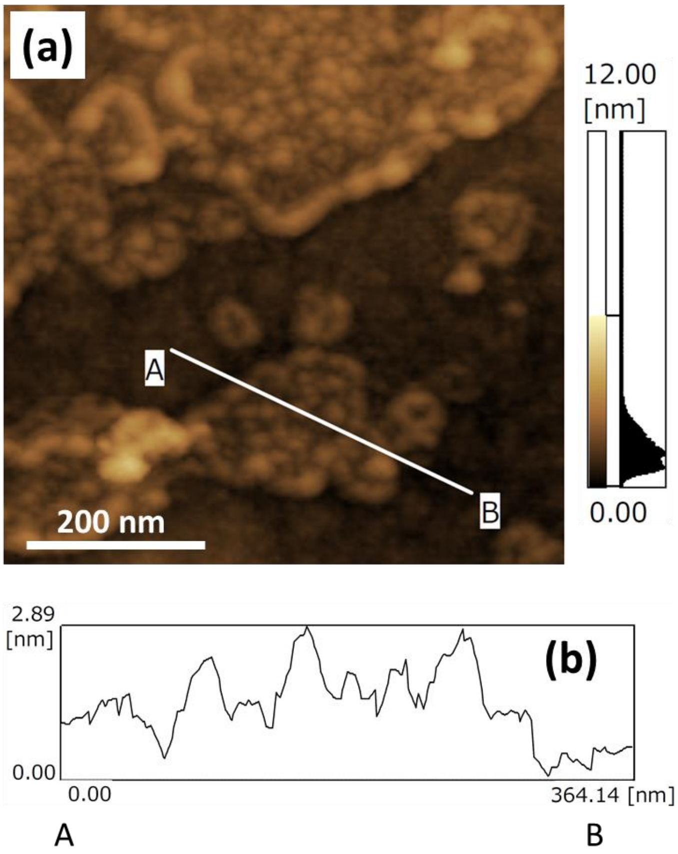

Figure S3. AFM image of HB nanosheets coated on an atomically flat mica substrate (a). Panel (b) shows the height profile of the line (A-B) indicated in panel (a).

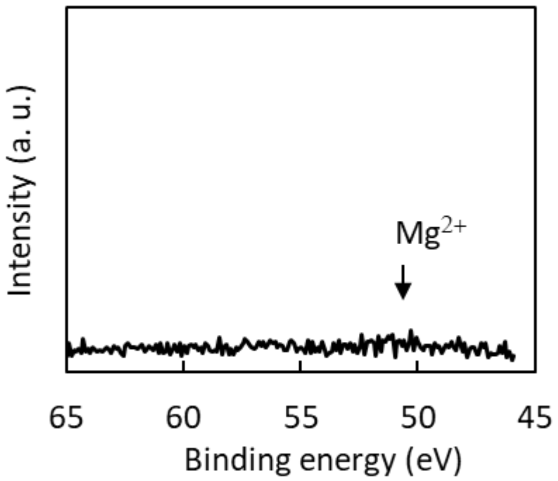

Figure S4. XPS spectrum of the magnesium 2p orbital for HB nanosheets.

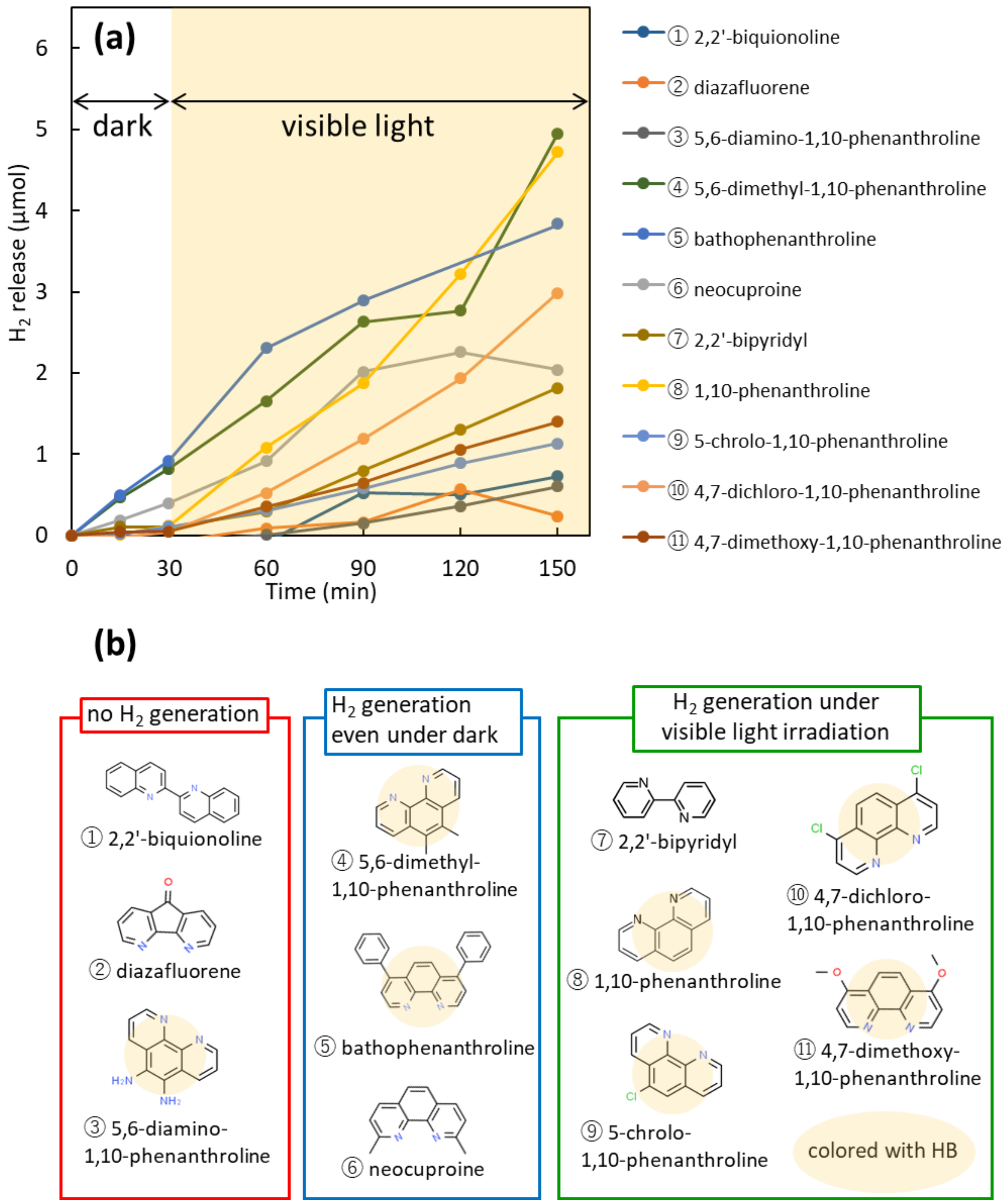

Figure S5. H_2 generation properties of various mixtures under visible light irradiation (a), molecular structures of nitrogen containing heterocycles categorized in three groups (b).

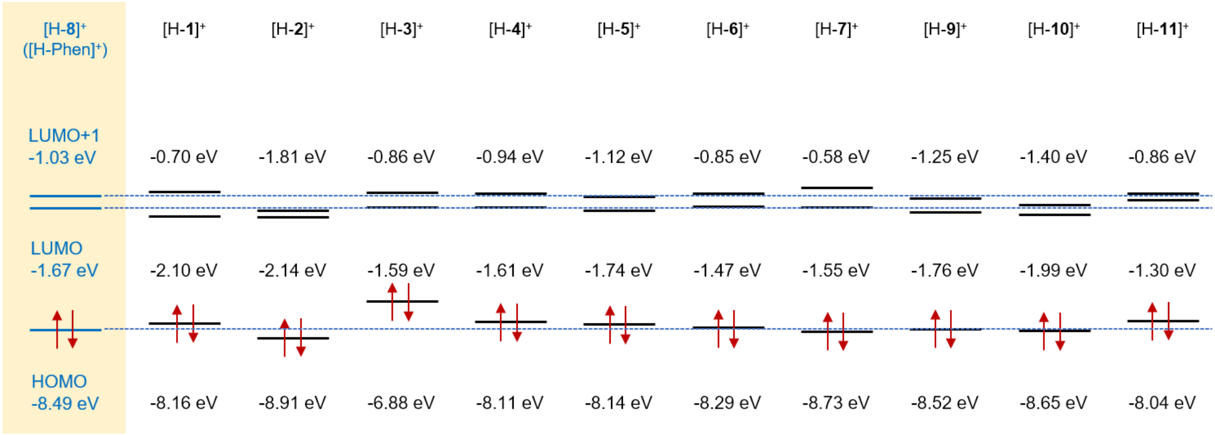

Figure S6. Electronic structure of various protonated heterocycles by DFT calculations. 1,10-phenanthroline (H-**8**), protonated 2,2'-biquionoline (H-**1**), protonated diazafluorene-9-on (H-**2**), protonated 5,6-diamino-1,10-phenanthroline (H-**3**), protonated 5,6-dimethyl-1,10-phenanthroline (H-**4**), protonated bathophenanthroline (H-**5**), protonated neocuproine (H-**6**), protonated 2,2'-bipyridyl (H-**7**), protonated 1,10-phenanthroline (H-**8**), protonated 5-chloro-1,10-phenanthroline (H-**9**), protonated 4,7-dichloro-1,10-phenanthroline (H-**10**), protonated 4,7-dimethoxy-1,10-phenanthroline (H-**11**).

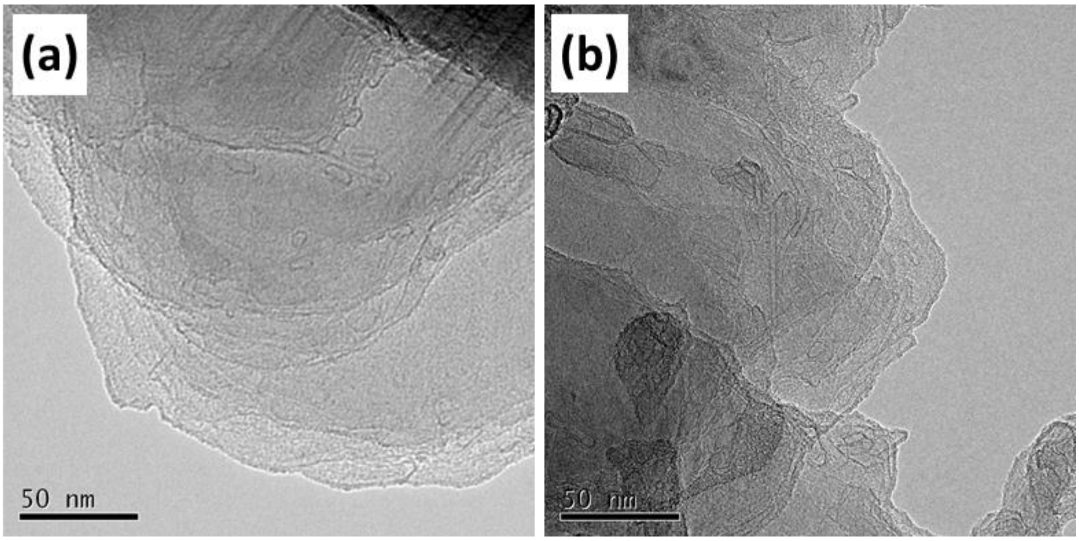

Figure S7. TEM images of HB+Phen before (a) and after visible light irradiation (b).

Note 3. DFT calculations method

Geometry optimizations were performed using the Gaussian16 program revision C.01,[1] without any symmetry constraints. Calculations were running using the restricted and unrestricted cam-B3LYP functional,[2] corrected for dispersion a proposed by Grimme (D3 correction with Becke-Johnson damping),[3] at the 6-311G+(d,p) basis set[4] with a frequency calculation, and the SMD solvation model[5] is used for CH_3CN. Thermodynamic corrections were calculated with frequency analysis to be either minima (with no imaginary frequencies) at 298.15 K. The cartesian coordinates are shown in the later section in this Supporting Information.

References in the section of DFT calculations,

1) Frisch, M. J.; Trucks, G. W.; Schlegel, H. B.; Scuseria, G. E.; Robb, M. A.; Cheeseman, J. R.; Scalmani, G.; Barone, V.; Petersson, G. A.; Nakatsuji, H.; Li, X.; Caricato, M.; Marenich, A. V.; Bloino, J.; Janesko, B. G.; Gomperts, R.; Mennucci, B.; Hratchian, H. P.; Ortiz, V. J.; Izmaylov, A. F.; Sonnenberg, J. L.; Williams-Young, D.; Ding, F.; Lipparini, F.; Egidi, F.; Goings, J.; Peng, B.; Petrone, A.; Henderson, T.; Ranasinghe, D.; Zakrzewski, V. G.; Gao, J.; Rega, N.; Zheng, G.; Liang, W.; Hada, M.; Ehara, M.; Toyota, K.; Fukuda, R.; Hasegawa, J.; Ishida, M.; Nakajima, T.; Honda, Y.; Kitao, O.; Nakai, H.; Vreven, T.; Throssell, K. Jr.; Montgomery, J. A.; Peralta, J. E.; Ogliaro, F.; Bearpark, M. J.; Heyd, J. J.; Brothers, E. N.; Kudin, K. N.; Staroverov, V. N.; Keith, T. A.; Kobayashi, R.; Normand, J.; Raghavachari, K.; Rendell, A. P.; Burant, J. C.; Iyengar, S. S.; Tomasi, J.; Cossi, M.; Millam, J. M.; Klene, M.; Adamo, C.; Cammi, R.; Ochterski, J. W.; Martin, R. L.; Morokuma, K.; Farkas, O.; Foresman, J. B.; Fox, D. J. Gaussian 16, Revision C.01; Gaussian, Inc., Wallingford CT, 2016.

2) Yanai, T.; Tew, D.; Handy, N. *Chem. Phys. Lett.*, **2004**, *393*, 51-57.

3) (a) Grimme, S.; Ehrlich, S.; Goerigk, L. J. *Comput. Chem.* **2011**, *32*, 1456-1465. (b) Grimme, S. Dispersion Interaction and Chemical Bonding. In *The Chemical Bond*, Wiley−VCH Verlag GmbH & Co. KGaA: Weinheim, Germany, **2014**, 477-500.

4) Krishnan, R.; Binkley, J. S.; Seeger, R.; Pople, J. A. *J. Chem. Phys.* **1980**, *72*, 650-654.

5) Marenich, A. V.; Cramer, C. J.; Truhlar, D. G. *J. Phys. Chem. B* **2009**, *113*, 6378−6396.

Cartesian coordinates of heterocyclic compounds

- Protonated 2,2'-biquionoline (H-**1**)

C	-5.19027	-4.78013	-0.00707
C	-3.78732	-4.91976	-0.04052
C	-3.61381	-2.54409	-0.00072
C	-5.72164	-3.46412	0.02322
C	-2.82846	-1.28666	0.00623
C	-0.83778	0.03437	0.04700
C	-1.60751	1.21478	0.01334
C	-3.01227	1.08893	-0.01963
H	-4.55841	-0.29209	-0.04112
N	-3.54168	-0.16708	-0.02139

N	-4.91579	-2.37762	0.02568
H	-3.35177	-5.91168	-0.06932
H	0.24237	0.11480	0.07747
C	-1.43322	-1.20019	0.04396
H	-0.83610	-2.09846	0.07618
C	-2.99175	-3.80918	-0.03840
H	-1.91602	-3.90263	-0.07124
C	-1.85848	3.60947	-0.01873
H	-1.43098	4.60415	-0.01949
C	-3.26295	3.46005	-0.05095
H	-3.88996	4.34275	-0.07567
C	-7.42720	-5.67911	0.02325
H	-8.10516	-6.52402	0.02433
C	-7.95478	-4.36580	0.05181
H	-9.02887	-4.22599	0.07384
C	-1.04481	2.51193	0.01327
H	0.03333	2.61153	0.03893
C	-3.84318	2.22055	-0.05142
H	-4.91855	2.09363	-0.07632
C	-6.07679	-5.88384	-0.00573
H	-5.66382	-6.88562	-0.02827
C	-7.12583	-3.28060	0.05221
H	-7.51312	-2.26927	0.07458

- Protonated diazafluorene-9-on (H-**2**)

C	-4.92163	-4.84579	-0.00384
C	-3.53992	-4.68442	-0.00495
C	-3.07145	-3.38625	-0.00319
C	-3.99175	-2.33852	-0.00049
C	-5.75138	-3.72903	-0.00106
C	-3.23407	-1.07592	0.00081
C	-1.87226	-1.32992	-0.00084
C	-0.97232	-0.28723	0.00004
C	-1.48049	1.01507	0.00273
C	-2.83904	1.22223	0.00426
H	-5.36336	-5.83347	-0.00509
H	-0.82051	1.87086	0.00356

H	-4.69379	0.34181	0.00450
N	-3.68766	0.17071	0.00327
N	-5.30177	-2.46657	0.00065
H	-3.29524	2.20152	0.00623
H	-6.82796	-3.85362	-0.00014
H	-2.86900	-5.53478	-0.00709
H	0.09589	-0.46667	-0.00128
C	-1.68702	-2.82455	-0.00368
O	-0.64225	-3.41869	-0.00597

- Protonated 5,6-diamino-1,10-phenanthroline (H-**3**)

C	-5.16359	-4.75143	0.01856
C	-3.79881	-4.86455	0.01263
C	-3.00264	-3.69913	-0.01429
C	-3.69625	-2.47760	-0.01780
C	-5.74957	-3.47468	-0.00281
C	-1.56470	-3.71522	-0.04386
C	-2.93723	-1.26898	-0.01435
C	-1.53511	-1.28100	0.02070
C	-0.84890	-2.53933	0.01698
C	-0.89180	-0.02628	0.05636
H	0.18740	0.03558	0.09793
C	-1.61203	1.14564	0.04054
C	-2.99774	1.09372	-0.00750
H	-5.79439	-5.63047	0.04234
H	-3.34824	-5.84815	0.04017
H	-6.82884	-3.36667	-0.00307
H	-1.11760	2.10599	0.06569
H	-3.63483	1.96635	-0.02498
H	-4.61553	-0.14873	-0.05628
N	-3.59612	-0.08824	-0.03013
N	-5.03887	-2.37035	-0.01701
N	0.54598	-2.60060	-0.00036
H	1.02663	-1.72674	0.15642
H	0.93663	-3.31799	0.60033
N	-0.88306	-4.92289	-0.06519
H	-1.39677	-5.71590	-0.42204
H	0.03902	-4.87803	-0.48196

- Protonated 5,6-dimethyl-1,10-phenanthroline (H-**4**)

C	-5.12874	-4.75768	0.02740
C	-3.76078	-4.85701	0.01828
C	-2.97391	-3.68677	0.00862
C	-3.68174	-2.47834	0.00907
C	-5.72513	-3.48860	0.02737
C	-1.52131	-3.70654	-0.00139
C	-2.92842	-1.26181	-0.00214
C	-1.53292	-1.27165	-0.01290
C	-0.81902	-2.53398	-0.01084
C	-0.89242	-0.01763	-0.02575
H	0.18741	0.03456	-0.03473
C	-1.61626	1.15551	-0.02725
C	-2.99942	1.09816	-0.01523
H	-5.75151	-5.64282	0.03448
H	-3.29216	-5.83100	0.01839
H	-6.80520	-3.38894	0.03470
H	-1.12469	2.11765	-0.03716
H	-3.64036	1.96843	-0.01493
H	-4.61570	-0.15137	0.00550
N	-3.59621	-0.08705	-0.00330
N	-5.02303	-2.37643	0.01854
C	0.68163	-2.45039	-0.01919
H	1.03358	-1.91435	-0.90449
H	1.04200	-1.90293	0.85569
H	1.15585	-3.42539	-0.01491
C	-0.87493	-5.06335	-0.00079
H	-1.17533	-5.63111	0.88350
H	-1.18989	-5.63844	-0.87521
H	0.20860	-5.01613	-0.01013

- Protonated bathophenanthroline (H-**5**)

C	-5.06380	-4.79753	-0.00615
C	-3.69426	-4.93309	-0.05631
C	-2.91328	-3.74616	-0.04391
C	-3.60663	-2.52592	-0.01813
C	-5.64801	-3.52440	0.02192

C	-1.47995	-3.72608	-0.03340
C	-2.84968	-1.30567	-0.01251
C	-1.45215	-1.29912	0.01304
C	-0.78370	-2.56689	-0.00761
C	-0.79018	-0.04148	0.03234
C	-1.55014	1.11640	-0.02578
C	-2.93003	1.04692	-0.05743
H	-5.70131	-5.67214	-0.01109
H	-6.72790	-3.42498	0.04816
H	-1.07588	2.08729	-0.01700
H	-3.56944	1.91808	-0.08422
H	-4.54758	-0.20486	-0.04655
N	-3.52865	-0.13630	-0.03730
N	-4.94711	-2.41223	0.00603
H	-0.94433	-4.66468	-0.04225
H	0.29601	-2.59060	-0.01058
C	0.68257	0.07054	0.11045
C	1.36575	0.84376	-0.82821
C	1.39434	-0.55084	1.13725
C	2.74453	0.97645	-0.75211
H	0.81686	1.32825	-1.62707
C	2.77070	-0.40102	1.21945
H	0.86886	-1.13400	1.88374
C	3.44885	0.35635	0.27221
H	3.26857	1.56736	-1.49368
H	3.31415	-0.87714	2.02666
H	4.52504	0.46581	0.33439
C	-3.08629	-6.28441	-0.11516
C	-2.23241	-6.64250	-1.15863
C	-3.39930	-7.22799	0.86266
C	-1.70033	-7.92253	-1.22014
H	-1.99682	-5.92207	-1.93290
C	-2.85727	-8.50463	0.80488
H	-4.06049	-6.95575	1.67703
C	-2.00757	-8.85492	-0.23662
H	-1.04571	-8.19244	-2.04033
H	-3.09998	-9.22636	1.57579
H	-1.58771	-9.85277	-0.28354

- Protonated neocuproine (H-**6**)

C	-5.12709	-4.76624	-0.10001
C	-3.76538	-4.85944	-0.09048
C	-2.99190	-3.67950	-0.05067
C	-3.70013	-2.47097	-0.02504
C	-5.75159	-3.49427	-0.07100
C	-1.56153	-3.67470	-0.03335
C	-2.94879	-1.25505	0.01723
C	-1.55506	-1.25942	0.03935
C	-0.86708	-2.51268	0.01117
C	-0.90367	-0.00905	0.08896
C	-1.62260	1.15674	0.10873
C	-3.02232	1.12098	0.07794
H	-5.74515	-5.65501	-0.12850
H	-1.13110	2.11913	0.14461
H	-4.63255	-0.13115	0.01575
N	-3.61289	-0.06947	0.03694
N	-5.04673	-2.38276	-0.03600
H	-1.04442	-4.62653	-0.05485
H	0.21567	-2.50595	0.02648
H	0.17950	0.01842	0.11003
H	-3.26969	-5.82293	-0.11162
C	-7.24386	-3.39209	-0.07633
H	-7.55560	-2.34873	-0.07729
H	-7.65811	-3.89080	-0.95602
H	-7.66161	-3.88802	0.80369
C	-3.86247	2.34607	0.08352
H	-4.92604	2.11020	0.08041
H	-3.62978	2.94491	0.96627
H	-3.62824	2.95061	-0.79544

- Protonated 2,2’-bipyridyl (H-**7**)

C	-5.21435	-4.73591	-0.00313
C	-3.84207	-4.91707	-0.00580
C	-3.60048	-2.54911	-0.00124
C	-5.71173	-3.43847	0.00034
C	-2.81546	-1.29278	0.00006

C	-0.86688	0.09041	0.00140
C	-1.66527	1.22753	0.00321
C	-3.03090	1.06902	0.00342
H	-5.89426	-5.57789	-0.00373
H	-1.23924	2.22053	0.00447
H	-4.56170	-0.30366	0.00225
N	-3.54672	-0.16429	0.00185
N	-4.92447	-2.37094	0.00124
H	-3.73738	1.88712	0.00477
H	-6.78017	-3.25348	0.00243
H	-3.41566	-5.91243	-0.00862
H	0.21142	0.18759	0.00127
C	-1.43758	-1.17383	-0.00024
H	-0.81081	-2.05319	-0.00147
C	-3.01154	-3.80528	-0.00489
H	-1.93783	-3.92762	-0.00717

- Protonated 1,10-phenanthroline (H-**8**)

C	-5.11912	-4.80387	-0.00364
C	-3.75005	-4.85777	-0.00513
C	-3.01128	-3.66131	-0.00380
C	-3.72473	-2.44503	-0.00093
C	-5.73542	-3.54446	-0.00081
C	-1.57780	-3.66276	-0.00521
C	-2.97999	-1.19487	0.00051
C	-1.57071	-1.24338	-0.00097
C	-0.88723	-2.50366	-0.00385
C	-0.87018	-0.02400	0.00040
H	0.21400	-0.03448	-0.00068
C	-1.56949	1.15423	0.00299
C	-2.97043	1.09671	0.00427
H	-1.06553	-4.61793	-0.00737
H	-5.72282	-5.70235	-0.00456
H	-3.22418	-5.80581	-0.00729
H	-6.81910	-3.47426	0.00042
H	0.19671	-2.49965	-0.00488
H	-1.06669	2.11280	0.00404
H	-3.54800	2.01629	0.00628

N	-3.65541	-0.02794	0.00312
N	-5.07236	-2.40677	0.00051

- Protonated 5-chloro-1,10-phenanthroline (H-**9**)

C	-5.13788	-4.76963	-0.00372
C	-3.77152	-4.87316	-0.00536
C	-2.99481	-3.70023	-0.00398
C	-3.69165	-2.47947	-0.00088
C	-5.73135	-3.49797	-0.00065
C	-1.55545	-3.66638	-0.00531
C	-2.94274	-1.25665	0.00061
C	-1.54817	-1.25805	-0.00083
C	-0.85813	-2.51108	-0.00380
C	-0.88663	-0.01707	0.00052
C	-1.60539	1.15657	0.00291
C	-2.99190	1.10123	0.00415
H	-5.76305	-5.65270	-0.00467
H	-1.11478	2.11916	0.00376
H	-4.62320	-0.12602	0.00392
N	-3.60286	-0.07690	0.00307
N	-5.03191	-2.38425	0.00073
H	0.22375	-2.50809	-0.00477
H	0.19662	0.00305	-0.00052
H	-3.29551	-5.84426	-0.00760
H	-3.62550	1.97701	0.00588
H	-6.81132	-3.39877	0.00068
Cl	-0.68349	-5.17717	-0.00861

- Protonated 4,7-dichloro-1,10-phenanthroline (H-**10**)

C	-5.13801	-4.76290	-0.00388
C	-3.77373	-4.86043	-0.00541
C	-2.97391	-3.69677	-0.00384
C	-3.67826	-2.48207	-0.00072
C	-5.71912	-3.48541	-0.00071
C	-1.54347	-3.68589	-0.00507
C	-2.92266	-1.26191	0.00077
C	-1.52520	-1.26634	-0.00074
C	-0.84855	-2.52622	-0.00352

C	-0.89007	-0.00361	0.00035
C	-1.61555	1.16642	0.00279
C	-2.99809	1.09220	0.00419
H	-5.76606	-5.64322	-0.00498
H	-1.13281	2.13278	0.00356
H	-4.61667	-0.15228	0.00411
N	-3.59643	-0.08974	0.00320
N	-5.01759	-2.37492	0.00081
H	0.23172	-2.54475	-0.00438
H	-3.63414	1.96616	0.00596
H	-6.79908	-3.38986	0.00053
H	-1.01041	-4.62653	-0.00708
Cl	0.83225	0.09189	-0.00176
Cl	-3.03047	-6.43050	-0.00926

- Protonated 4,7-dimethoxy-1,10-phenanthroline (H-**11**)

C	-5.21030	-4.72897	-0.00473
C	-3.83551	-4.83031	-0.00586
C	-3.06254	-3.63543	-0.00445
C	-3.77013	-2.42760	-0.00143
C	-5.79387	-3.45549	-0.00189
C	-1.63546	-3.63426	-0.00573
C	-3.01525	-1.20994	0.00050
C	-1.62521	-1.22021	-0.00074
C	-0.93919	-2.47123	-0.00395
C	-0.95066	0.03691	0.00117
C	-1.68835	1.21666	0.00411
C	-3.06431	1.14474	0.00520
H	-5.84817	-5.60041	-0.00587
H	-1.21718	2.18678	0.00564
H	-4.70448	-0.07966	0.00419
N	-3.68666	-0.02901	0.00345
N	-5.11579	-2.32801	-0.00015
H	0.14172	-2.47294	-0.00489
H	-3.69099	2.02570	0.00743
H	-6.87589	-3.37378	-0.00097
H	-1.11551	-4.58247	-0.00806
O	0.36473	-0.01716	-0.00011

O	-3.14850	-5.97231	-0.00809
C	1.12547	1.20287	0.00092
H	2.16621	0.89220	-0.00048
H	0.90698	1.78432	-0.89510
H	0.90865	1.78180	0.89897
C	-3.87096	-7.20734	-0.00817
H	-4.49094	-7.28991	-0.90274
H	-3.11532	-7.98881	-0.00936
H	-4.48923	-7.29096	0.88748

- 1,10-phenanthroline (**8**)

C	-5.11912	-4.80387	-0.00364
C	-3.75005	-4.85777	-0.00513
C	-3.01128	-3.66131	-0.00380
C	-3.72473	-2.44503	-0.00093
C	-5.73542	-3.54446	-0.00081
C	-1.57780	-3.66276	-0.00521
C	-2.97999	-1.19487	0.00051
C	-1.57071	-1.24338	-0.00097
C	-0.88723	-2.50366	-0.00385
C	-0.87018	-0.02400	0.00040
H	0.21400	-0.03448	-0.00068
C	-1.56949	1.15423	0.00299
C	-2.97043	1.09671	0.00427
H	-1.06553	-4.61793	-0.00737
H	-5.72282	-5.70235	-0.00456
H	-3.22418	-5.80581	-0.00729
H	-6.81910	-3.47426	0.00042
H	0.19671	-2.49965	-0.00488
H	-1.06669	2.11280	0.00404
H	-3.54800	2.01629	0.00628
N	-3.65541	-0.02794	0.00312
N	-5.07236	-2.40677	0.00051

- Singly-reduced protonated 1,10-phenanthroline ([H-**8**]$^{\bullet}$)

C	-5.12789	-4.79492	-0.00367
C	-3.75818	-4.87921	-0.00518
C	-2.99004	-3.70247	-0.00386

C	-3.68859	-2.46694	-0.00099
C	-5.72583	-3.52079	-0.00080
C	-1.56853	-3.69418	-0.00522
C	-2.95502	-1.25764	0.00037
C	-1.55351	-1.25594	-0.00101
C	-0.88346	-2.51869	-0.00384
C	-0.86459	-0.01648	0.00040
H	0.21738	-0.00325	-0.00062
C	-1.60865	1.18302	0.00301
C	-2.97008	1.15485	0.00431
H	-1.04170	-4.64157	-0.00737
H	-5.75017	-5.68042	-0.00461
H	-3.25632	-5.84061	-0.00738
H	-6.80869	-3.43622	0.00044
H	0.20072	-2.51760	-0.00487
H	-1.10673	2.14213	0.00403
H	-3.59262	2.03771	0.00625
H	-4.64321	-0.08954	0.00391
N	-3.63332	-0.05297	0.00307
N	-5.04816	-2.39553	0.00052
